# The circadian clock component *NPAS2* is modulated by AMPK and regulates substrate utilization and mitochondrial function in human skeletal muscle cells

**DOI:** 10.1016/j.molmet.2026.102446

**Published:** 2026-09-19

**Authors:** David Rizo-Roca, Logan A. Pendergrast, Prasad Kamble, Dimitrius Santiago P.S.F. Guimarães, Lucile Dollet, Marie Björnholm, Juleen R. Zierath

**Affiliations:** 1 Department of Physiology and Pharmacology, Section for Integrative Physiology, Karolinska Institutet, Stockholm, Sweden; 2 Department of Molecular Medicine and Surgery, Section for Integrative Physiology, Karolinska Institutet, Stockholm, Sweden; 3 Novo Nordisk Foundation Center for Basic Metabolic Research, University of Copenhagen, Copenhagen, Denmark

**Keywords:** Skeletal muscle, Circadian clock, Metabolic regulation, Glucose and lipid metabolism

## Abstract

The molecular circadian clock coordinates cellular and systemic metabolism across the day, yet how it integrates with energy-sensing pathways in human skeletal muscle remains incompletely understood. AMP-activated protein kinase (AMPK) functions as a cellular energy sensor and has been implicated in circadian regulation; however, its role in coupling clock components to metabolic programming in human muscle has not been established.

Here, we investigated the interaction between AMPK signaling and the circadian machinery in primary human myotubes, focusing on the downstream clock components NPAS2 and DBP. Silencing of the AMPKα subunit altered core clock gene expression, increasing *CRY1* and *CRY2* while reducing *NPAS2* and *DBP* expression, with rhythmicity preserved. To determine the functional relevance of these changes, we examined metabolic phenotypes following siRNA-mediated silencing of *NPAS2*. *NPAS2* knockdown reduced basal oxidative metabolism, decreased abundance of electron transport chain subunits, and increased β-oxidation flux, while maximal mitochondrial respiratory capacity remained intact. These alterations occurred independently of canonical AMPK–ACC signaling but were associated with reduced *HIF1A* expression. In contrast, *DBP* silencing reduced glucose oxidation but did not recapitulate the broader metabolic remodeling observed with *NPAS2* knockdown.

Together, these findings identify NPAS2 as a downstream mediator linking AMPK-dependent circadian signaling to skeletal muscle metabolic regulation. Disruption of this axis alters substrate utilization and impairs mitochondrial function, revealing a mechanism by which circadian clock components contribute to metabolic regulation in human skeletal muscle.

## Highlights

• AMPKα silencing reduces *NPAS2* and *DBP* while increasing *CRY1* and *CRY2*.
• NPAS2 knockdown lowers oxidative metabolism.
• NPAS2 silencing increases β-oxidation.
• DBP silencing reduces glucose oxidation without broader metabolic remodeling.

## 1 Introduction

The molecular circadian clock governs metabolic homeostasis and is present in nearly all cells of the body, operating through a transcription-translation feedback loop initiated by heterodimerization of the transcription factors brain and muscle ARNT-like 1 (BMAL1), circadian locomotor output cycles kaput (CLOCK); and neuronal PAS domain protein 2 (NPAS2). This transcriptional process drives expression of the repressor proteins period (PER) and cryptochrome (CRY), which, upon translation, translocate to the nucleus and repress the clock transcription factor heterodimers [1]. The core molecular clock also drives rhythmic expression of clock-controlled genes, including albumin D-site-binding protein (DBP), a transcription factor that relays circadian information to downstream targets, such as PER1, thereby contributing to fine-tuning circadian gene networks [2]. In peripheral tissues, circadian clocks coordinate expression of genes involved in glucose and energy homeostasis, enabling temporal organization of metabolic processes across the day [3,4]. Disruption of this system is associated with metabolic diseases, including impaired lipid and glucose homeostasis in type 2 diabetes [5,6].

Circadian timing in peripheral tissues is regulated by systemic and local signals, including nutrients, hormones, and cellular energy status, promoting coherent and efficient temporal gating of metabolic processes [[7], [8], [9], [10], [11]]. Among these, AMP-activated protein kinase (AMPK) functions as a key cellular energy sensor that promotes catabolic metabolism and coordinates glucose and lipid utilization [[12], [13], [14]]. AMPK also interfaces with the circadian clock: AMPK activation destabilizes CRY proteins and alters clock gene expression in cultured mouse fibroblasts [15] and in mouse skeletal muscle [16], while circadian transcription factors, including the basic helix-loop-helix transcription factor differentiated embryo-chondrocyte 1 (DEC1), and oscillatory cAMP-protein kinase A signaling reciprocally modulate AMPK activity [17,18]. Together, these findings indicate reciprocal regulation between the circadian molecular clock and AMPK.

The mechanism by which AMPK regulates specific skeletal muscle clock components and influences metabolic programming remain incompletely understood. In particular, whether AMPK signaling is required to maintain expression of core clock genes such as *NPAS2* and *NPAS2*-dependent downstream metabolic programs in human skeletal muscle remains unresolved. Here, we test the hypothesis that AMPKα regulates skeletal muscle circadian gene expression and investigate whether AMPK-regulated clock components influence downstream metabolic programming, focusing on NPAS2 and DBP as representative core and clock-controlled components linking energy sensing to metabolism.

## 2 Methods and materials

### 2.1 Human primary muscle cell culture

Primary cells were obtained from *vastus lateralis* biopsies collected from 7 male donors as approved by the Ethical Committee in Stockholm. Clinical characteristics of the donors are shown in Table 1. Myoblasts were proliferated in growth medium (F12/DMEM, 25 mM glucose; no. 31331; Thermo Fisher Scientific) supplemented with 100 U/mL penicillin, 100 μg/mL of streptomycin, and 0.25 μg/mL of amphotericin B and 20% fetal bovine serum (FBS). Differentiation was performed in DMEM with GlutaMAX (no. 31966; Gibco), supplemented with 20% medium 199 (no. 31150; Gibco), HEPES buffer (0.02 M; no. 15630; Gibco), zinc sulfate (0.03 μg/mL), vitamin B_12_ (1.4 μg/mL; Sigma–Aldrich), insulin (10 μg/mL; Actrapid; Novo Nordisk), and apo-transferrin (100 μg/mL; no. T100-5; BBI Solutions). On day 4, cultures were switched to post-fusion medium, containing DMEM/M199, HEPES, zinc sulfate, vitamin B_12_, and 2% FBS until day 6. Differentiation was evaluated microscopically and by the analysis of myogenic markers (Fig. S1a).

**Table 1:** Clinical characteristics of primary skeletal muscle cell donors.

Clinical characteristic	Mean		SEM
Age	55	±	3.68
Weight [kg]	80	±	4.06
Length [m]	1.83	±	0.03
BMI [kg/m^2^]	23.96	±	0.89
Waist [cm]	90.0	±	3.30
Hip [cm]	98.3	±	2.28
Waist/Hip ratio	0.91	±	0.02
Fasting glucose [mmol/L]	5.4	±	0.23
HbA1c [mmol/mol]	34.7	±	1.04
S-insulin [mIE/L]	17.1	±	5.23
S–C-peptide [nmol/L]	0.55	±	0.08
P-creatinine [μmol/L]	80.3	±	3.40
P-ASAT [μkat/L]	0.36	±	0.02
P-ALAT [μkat/L]	0.36	±	0.03
P-TG [mmol/L]	1.00	±	0.24
P-cholesterol [mmol/L]	5.47	±	0.42
P-HDL [mmol/L]	1.31	±	0.14
P-LDL [mmol/L]	3.69	±	0.35

### 2.2 siRNA transfection and synchronization

On day 6 of differentiation, myotubes were simultaneously treated with predesigned validated Silencer Select siRNAs towards protein kinase AMP-activated catalytic subunit alpha 1 (5 nM, *PRKAA1*; no. 4390824, Thermo Fisher) and protein kinase AMP-activated catalytic subunit alpha 2 (*PRKAA2*) (5 nM; no. 4392420, Thermo Fisher), *DBP* (10 nM; no. 114134, Thermo Fisher), *NPAS2* (10 nM; no. 106844, Thermo Fisher), or Negative control No. 2 (10 nM; no. 4390847, Thermo Fisher). Each siRNA was diluted to 10 nM in OPTI-MEM (50 μL), then combined with Lipofectamine 2000 (Invitrogen, Sweden) diluted in OPTI-MEM according to the manufacturer's instructions. The reagent mixtures were combined and placed on a mechanical agitator for 30 min. For transfection, myotubes were rinsed twice with PBS, and 1 mL of DMEM was added per well. Next, 100 μl of siRNA transfection complex was added to the media for 5 h. Myotubes were then washed twice with PBS and maintained in growth medium until day 8, at which point this process was repeated identically. Serum shock was performed utilizing 50% serum DMEM, with samples collected in 6-hour intervals from 24 to 54 h post-shock (Figure 1A). For *DBP* and *NPAS2*-siRNA-treated cells, serum shock was performed using 50% serum DMEM, with samples collected at 48 h post-shock, as AMPKα knockdown effects on circadian gene expression were generally most robust at this time point while minimizing potential acute effects of the synchronization procedure.

**Figure 1:**
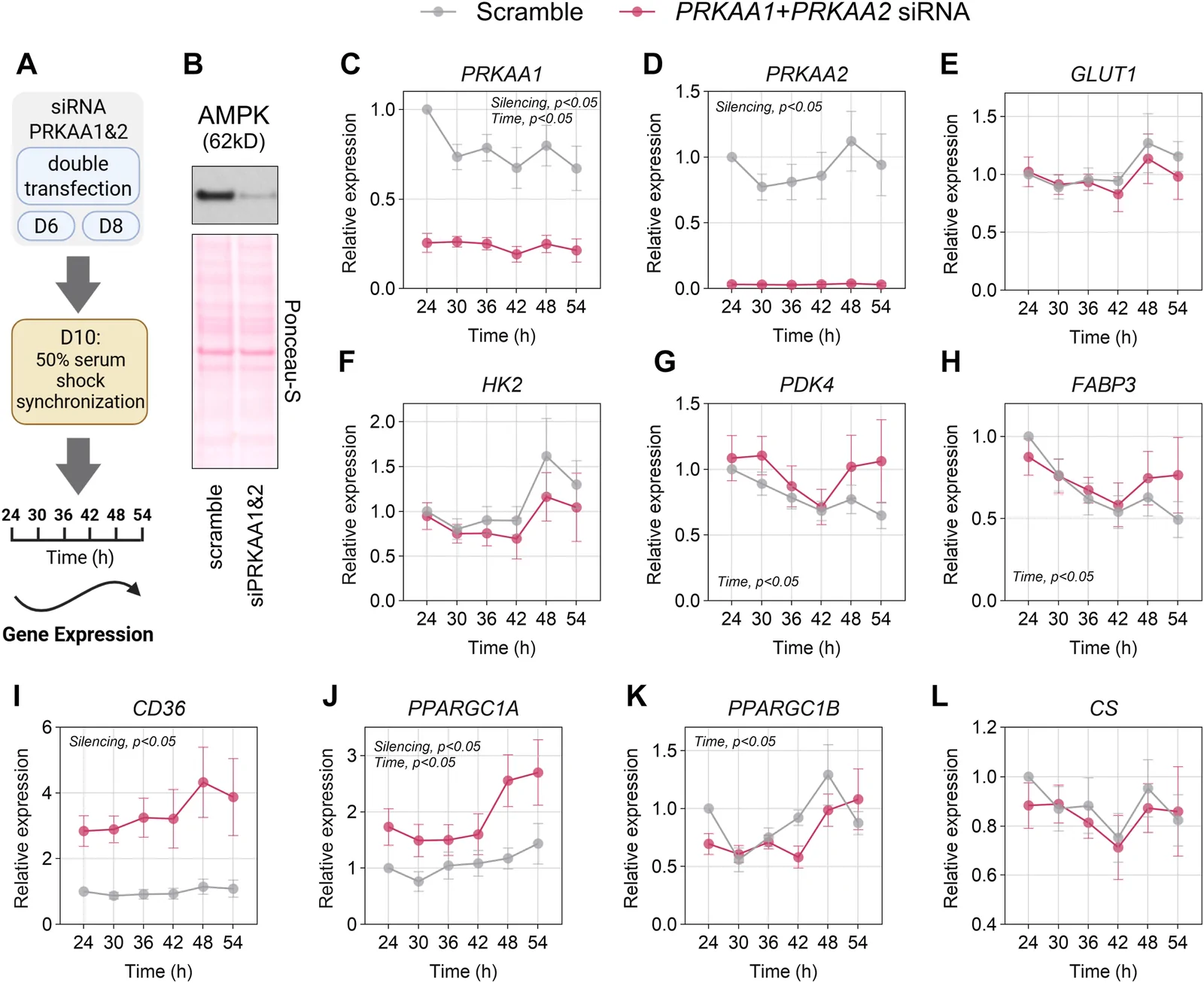
Effects of AMPKα silencing on the expression of metabolic genes in human skeletal muscle cells (n = 5). A) Experimental design. B) Representative immunoblot showing *PRKAA1* and *PRKAA2* silencing efficiency 48 h after serum shock synchronization. C) Silencing efficiency of *PRKAA1* (AMPKα1) from circadian time (CT) 24–54 h. D) Silencing efficiency *PRKAA2* gene (AMPKα2) over the same time course. E-L) Expression of *SLC2A1* (GLUT1) (E), *HK2* (F), *PDK4* (G), *FABP3* (H), *CD36* (I), *PPARGC1A* (J), *PPARGC1B* (K), and *CS* (L) following synchronization in *PRKAA1/PRKAA2* siRNA and scramble control siRNA-treated human skeletal muscle cells. Data were analyzed via two-way repeated-measures ANOVA (main effects of siRNA and time). Data are presented as mean ± SEM.

### 2.3 Palmitate oxidation assay

At 48 h post synchronization, myotubes treated with *DBP*-siRNA, *NPAS2*-siRNA, or scramble control siRNA were serum-starved for 1 h in low-glucose DMEM (5.5 mM). Myotubes were then incubated for 6 h in 1 mL of low-glucose DMEM supplemented with 25 μM palmitate, including radioactive palmitate ([9,10-^3^H(N)] palmitate) at a concentration of 0.078 μM (specific activity of 5 mCi/mL and 53.7 Ci/mmol). After incubation, the supernatant was collected and mixed with 0.8 mL of charcoal buffer (10% activated charcoal in a 0.02 M Tris–HCl solution, adjusted to a pH of 7.5) and shaken for 30 min. Samples were subjected to centrifugation (13,000 g for 15 min), and 0.2 mL of the cleared supernatant was taken for analysis by liquid scintillation counting. Myotubes were processed separately for protein determination using a Bradford assay.

### 2.4 Glucose oxidation assay

At 48 h post synchronization, myotubes treated with *DBP*-siRNA, *NPAS2*-siRNA, or scrambled control siRNA were serum-starved for 1 h in low-glucose DMEM (5.5 mM). Myotubes were then incubated with 2 mCi/mL d-[U–^14^C] glucose in the absence or presence of 120 nM insulin. A small empty cup was placed onto the medium in each well, after which the wells were sealed with a plastic film and incubated for 4 h at 37 °C in 0% CO_2_. Subsequently, [^14^C]-CO_2_ was liberated from the medium by addition of 150 μL of 2 M HCl and trapped by addition of 300 μL of 2 M NaOH, followed by a further 1-hour incubation at 37 °C in 0% CO_2_. Trapped [^14^C]-CO_2_ was quantified by liquid scintillation counting. Cells were then lysed in 0.5 M NaOH, neutralized with 2 M HCl, and protein content was determined using a BCA protein assay.

### 2.5 Lactate assay in cell media

Cell supernatant was collected from all silencing conditions at 48 h post-synchronization. Lactate concentration in the medium was determined using a colorimetric assay [19], with modifications as described [20].

### 2.6 Determination of gene expression

RNA was isolated using a trizol-chloroform extraction procedure (Fisher Scientific) [21] and cDNA was synthesized using a High-Capacity cDNA Reverse Transcription Kit (Thermo Fisher). Quantitative polymerase chain reaction (qPCR) was performed on a ViiA 7 Real-Time PCR System (Applied Biosciences) using TaqMan Fast Universal PCR Master Mix (Thermo Fisher). Cycling conditions were 95 °C for 40 cycles for 1 s and 60 °C for 20 s for 40 cycles. Cycle threshold was analyzed using Quantstudio Real-time PCR software (Thermo Fisher; version 1.3). All results were normalized to expression of the ribosomal subunit (18 S).

### 2.7 Determination of protein abundance

Protein abundance was determined by Western blot. Cells were lysed in radioimmunoprecipitation assay (RIPA) buffer (Thermo Fisher Scientific) and homogenized by sonication. Lysates were clarified by centrifugation at 16,000×*g*, and protein concentration in the supernatant was determined using a Bradford assay. Samples were prepared in Laemmli buffer and resolved in duplicates by SDS–polyacrylamide gel electrophoresis on 4–12% gradient gels. Proteins were transferred onto polyvinylidene difluoride (PVDF) membranes by wet transfer and stained with Ponceau S to verify transfer efficiency and for subsequent normalization. Membranes were blocked for 1 h in 5% skimmed milk in TBS-T [20 mM Tris–HCl, 150 mM NaCl, 0.02% (v/v) Tween 20, pH 7.5], followed by overnight incubation with diluted primary antibodies (1:1000) against ACC (Cell Signaling Technology, #3662 S, RRID), phospho-ACC^Ser79^ (Cell Signaling Technology, #3661, RRID:AB_330337), AMPK alpha (Cell Signaling Technology, #2532, RRID:AB_330331), phospho-AMPK^Thr172^ (Cell Signaling Technology, #2531, RRID:AB_330330), CPT1B (abcepta, #AP2532b, RRID:AB_2292207), GLUT1 (Merck, CBL242, RRID:AB_327046), and subunits of the electron transport chain (Total OXPHOS human WB Antibody Cocktail, abcam, ab110411, RRID:AB_2756818). After washing, membranes were incubated for 1 h with the appropriate horseradish peroxidase (HRP)-conjugated secondary antibodies (1:25,000). Signals were detected by enhanced chemiluminescence and visualized on X-ray film, which was subsequently scanned using a GS-800 Densitometer (Bio-Rad). After blot analysis for phospho-AMPK^Thr172^ and phospho-ACC^Ser79^, membranes were stripped and re-probed with additional antibodies against total AMPK and total ACC. Band intensities were quantified using ImageLab software (Bio-Rad).

Due to the number of experimental conditions, duplicate samples were analyzed across two membranes. All conditions from the same donor were loaded on the same gel to preserve paired comparisons. Band intensities were normalized to the corresponding lane Ponceau staining and subsequently median-centered within each membrane by dividing each normalized value by the median normalized intensity of all samples on that membrane. This procedure standardized signal intensity across membranes, while preserving relative differences between samples. Median-centered values were used for statistical analyses.

### 2.8 Seahorse XF96 bioenergetic assay

Mitochondrial function was assessed by an extracellular flux analyzer (Seahorse XFe96, Agilent), which measures both oxygen consumption rate (OCR) and extracellular acidification rate (ECAR). Myoblasts (5 × 10^3^ per well) were seeded in 96-well Seahorse plates, differentiated, transfected with the indicated siRNAs as described above, and synchronized. Plates were loaded into the instrument following a 40-minute equilibration in Seahorse DMEM (Agilent #103575–100) supplemented with 5.5 mM d-Glucose, 1X, GlutaMAX (Gibco #35050061) and 1 mM sodium pyruvate. Mitochondrial parameters were assessed by sequential injection of 1 μM oligomycin, 4 μM FCCP (added in two steps) and 0.5 μM rotenone/0.5 μM antimycin. For normalization, cells were fixed with 5% trichloroacetic acid (TCA) and stained with sulforhodamine B; the dye was resuspended in 20 mM Tris base, and absorbance was read at 565 nm. Rotenone and antimycin A, which inhibit electron transfer through complexes I and III, respectively, were used to calculate non-mitochondrial OCR, which was subtracted from all other parameters. The oligomycin-induced decrease in OCR was used to calculate ATP-linked respiration and proton leak. Maximal respiration was induced by the uncoupler FCCP. The FCCP-induced peak OCR was used to calculate spare respiratory capacity and maximal OCR.

### 2.9 Statistical analysis

Data are presented as mean ± standard error of the mean (SEM). Analyses were performed using one- (main effect of siRNA) or two-way repeated measures ANOVA (main effects of siRNA and time) followed by Dunnett's or Tukey's test, respectively. Data from experiments with n = 3 were analyzed using Friedman's test followed by Dunn's post-hoc test. Rhythmic oscillations in gene expression were analyzed using the RAIN package in R (Rhythmicity Analysis Incorporating Non-parametric Methods, release 3.15) [22], with period set to 24 h, and period delta set to 12 h. Outcomes with *P* < 0.05 were considered rhythmic.

## 3 Results

### 3.1 Effects of AMPK silencing on expression of genes involved in energy metabolism

To characterize the influence of AMPK on the circadian expression of metabolic genes, primary human skeletal muscle cells were doubly transfected with siRNA targeting *PRKAA1* and *PRKAA2* (encoding AMPKα1 and AMPKα2, respectively) and subsequently synchronized via serum shock (Figure 1A). Silencing was validated by Western blot (Figure 1B) and, at mRNA level, efficiency for *PRKAA1* ranged from 60 to 80% across all time points from circadian time (CT) 24–54 h (time after serum shock; Figure 1C), while silencing efficiency for *PRKAA2* exceeded 90% across all time points (Figure 1D). This degree of knockdown was sufficient to achieve sustained AMPKα suppression exceeding the magnitude of diurnal oscillation. We next evaluated the expression of genes central to glucose and fatty acid metabolism. Expression of glucose metabolism markers, including solute carrier family 2 member 1 (*SLC2A1*, encoding GLUT1) and hexokinase 2 (*HK2*), was unaffected by AMPKα silencing (Figure 1E–F). Similarly, pyruvate dehydrogenase kinase 4 (*PDK4*), an enzyme involved in regulating substrate preference, varied with time but was not affected by *PRKAA1/2*-silencing (Figure 1G). Fatty acid binding protein 3 (*FABP3*) expression also varied with time, independent of AMPKα silencing (Figure 1H). In contrast, *PRKAA1/2*-silencing increased fatty acid transporter *CD3*6 mRNA expression by approximately 300% (Figure 1I). Expression of peroxisome proliferator-activated receptor gamma coactivator 1-alpha (*PPARGC1A*) was increased in *PRKAA1/2*-silenced myotubes, with additional time-dependent variation (Figure 1J); this increase may partially explain the elevated *CD36* expression, as the encoded protein, PGC-1α, mediates the expression of genes involved in fatty acid oxidation. Expression of the *PPARGC1A* paralogue, peroxisome proliferator-activated receptor gamma coactivator 1-beta (*PPARGC1B*), was unaltered (Figure 1K). Despite the increased expression of *PPARGC1A*, citrate synthase (*CS*) expression was unaffected by *PRKAA1/2*-silencing, suggesting that mitochondrial content was not altered (Figure 1L).

### 3.2 Effects of AMPK silencing on circadian gene expression

AMPKα silencing altered expression of core clock genes across the time course studied. Successful synchronization was confirmed for most clock genes in the scramble control condition, as *DBP*, *BMAL1*, *CRY1*, *CRY2*, nuclear receptor subfamily 1 group D member 1 (*NR1D1*), *PER1*, *PER2*, and *PER3* all displayed rhythmicity, as determined using RAIN package in R [22]. In *PRKAA1/2*-silenced myotubes, only *NR1D1* lost rhythmicity. Expression of the transcriptional clock activators *CLOCK*, *NPAS2*, and *DBP* was reduced in *PRKAA1/2*-silenced myotubes (Figure 2A–C), suggesting that AMPK influences regulation of molecular clock function. Gene silencing had no effect on the transcriptional clock activator *BMAL1* (Figure 2D). Expression of the transcriptional clock repressors *CRY1* and *CRY2* was increased in *PRKAA1/2*-silenced myotubes (Figure 2E–F), consistent with a previous reported interaction between AMPK and CRY [15]. Expression of *NR1D1* and *PER1-3* was unaffected by *PRKAA1/2*-silencing (Figure 2G–J). We next investigated whether the clock genes downregulated by AMPKα silencing, *NPAS2* and *DBP*, were associated with the expression of metabolic genes (Figure 2K). *NPAS2* expression was positively associated with *PPARGC1A* and *CS* mRNA levels (Figure 2L-M), whereas *DBP* as negatively correlated with *PPARGC1A* and *CD36* expression (Figure 2N-O).

**Figure 2:**
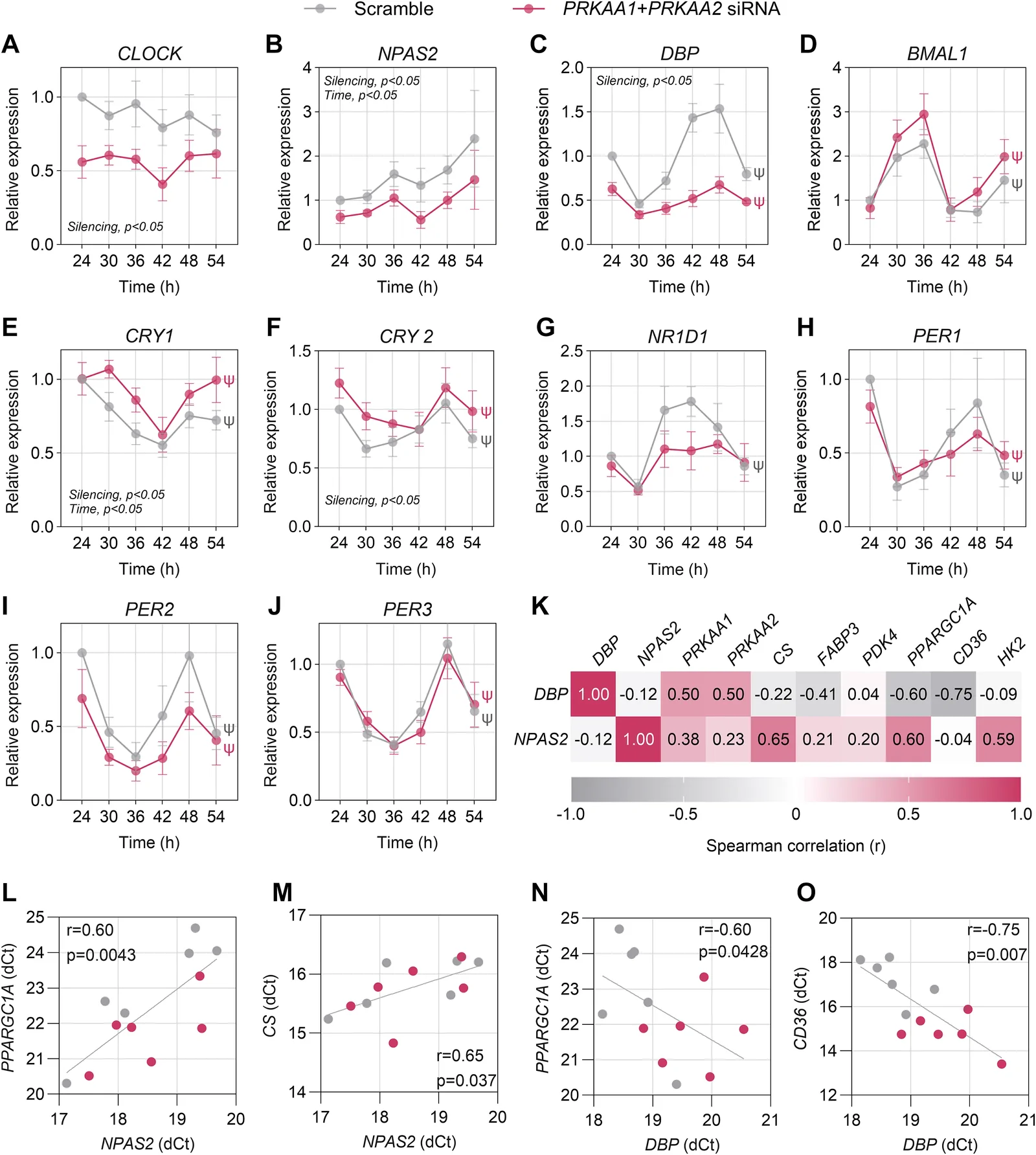
Characterization of *PRKAA1/PRKAA2* silencing on circadian gene expression in human skeletal muscle cells (n = 5). A-J) Expression of *CLOCK* (A), *NPAS2* (B), *DBP* (C), *BMAL1* (D), *CRY1* (E), *CRY2* (F), *NR1D1* (G), *PER1* (H), *PER2* (I), and *PER3* (J) following synchronization in *PRKAA1/PRKAA2* siRNA and scramble control siRNA-treated human skeletal muscle cells. K) Heatmap showing spearman correlation coefficients between *DBP*, *NPAS2*, and metabolic genes 48 h post-synchronization. L-M) Correlation between *NPAS2* and *PPARGC1A* (L) and *CS* (M). N-O) Correlation between *DBP* and *PPARGC1A* (N) and *CD36* (O). ψ Rhythmic expression pattern as identified by RAIN analysis: P < 0.05. Data were analyzed via two-way repeated-measures ANOVA (main effects of siRNA and time). Data are presented as mean ± SEM.

### 3.3 Effect of gene silencing on glucose and palmitate oxidation

To characterize the influence of *DBP* and *NPAS2* on glucose and lipid metabolism in human skeletal muscle cells, we transfected primary human myotubes with siRNA targeting either *DBP* or *NPAS2*. Myotubes were synchronized via serum shock, and gene expression and substrate utilization were subsequently determined. Expression of *DBP* and *NPAS2* was effectively downregulated by the respective siRNAs (Figure 3A–B). In *DBP*-silenced myotubes, expression of both *PRKAA1* and *PRKAA2* was increased (Figure 3C–D), suggesting a bidirectional transcriptional relationship between DBP and AMPK; this effect did not reach statistical significance in *NPAS2*-silenced myotubes. Nevertheless, these transcriptional alterations did not translate into changes at the protein level, as assessed by Western blot 48 h post-synchronization (Figure 3E–F). Expression of genes involved in glucose metabolism was unaffected by any of the silencing conditions (Figure 3G–I), although protein content of the glucose transporter GLUT1 was increased in *NPAS2*-silenced myotubes (Figure 3J). Despite the absence of changes in AMPK or related metabolic gene expression, glucose oxidation rates were reduced under both basal and insulin-stimulated conditions (Figure 3K), despite increased GLUT1 protein abundance, and lactate production was unchanged (Figure 3L). Collectively, these data suggest that DBP and NPAS2 may regulate oxidative metabolism independently of detectable alterations in proximal AMPK signaling. We therefore assessed whether *DBP* and *NPAS2* silencing altered substrate partitioning by enhancing fatty acid metabolism. Expression and phosphorylation of the AMPK downstream target acetyl-CoA carboxylase alpha (ACC) as well as the rate-limiting step of mitochondrial β-oxidation, carnitine palmitoyltransferase 1B (CPT1B), were unaffected by *DBP* and *NPAS2* silencing (Figure 3M-O). Nevertheless, relative mRNA levels of *FABP3* and *CD36* were upregulated in *NPAS2*- and *DBP*-silenced myotubes, respectively (Figure 3P-Q), whereas expression of *ACAA2* (Figure 3R), *PPARA* (Figure 3R), and *RORA* (Figure 3T), genes involved in fatty acid oxidation and circadian-metabolic regulation, was reduced. Furthermore, hypoxia inducible factor 1 subunit alpha (*HIF1A*) was downregulated upon *NPAS2* silencing (Figure 3U). NPAS2 interacts with HIF-1α, a transcription factor that reprograms fuel preference towards glycolytic energy metabolism and suppresses fatty acid oxidation [23,24]. Consistent with these findings, β-oxidation flux increased following *NPAS2* silencing (Figure 3V). Together with the observed reduction in glucose oxidation, these findings suggest that DBP and NPAS2 differentially impact substrate utilization (Figure 3W), at least in part independently of the AMPK-ACC axis (Figure 3E–F, M-O, X).

**Figure 3:**
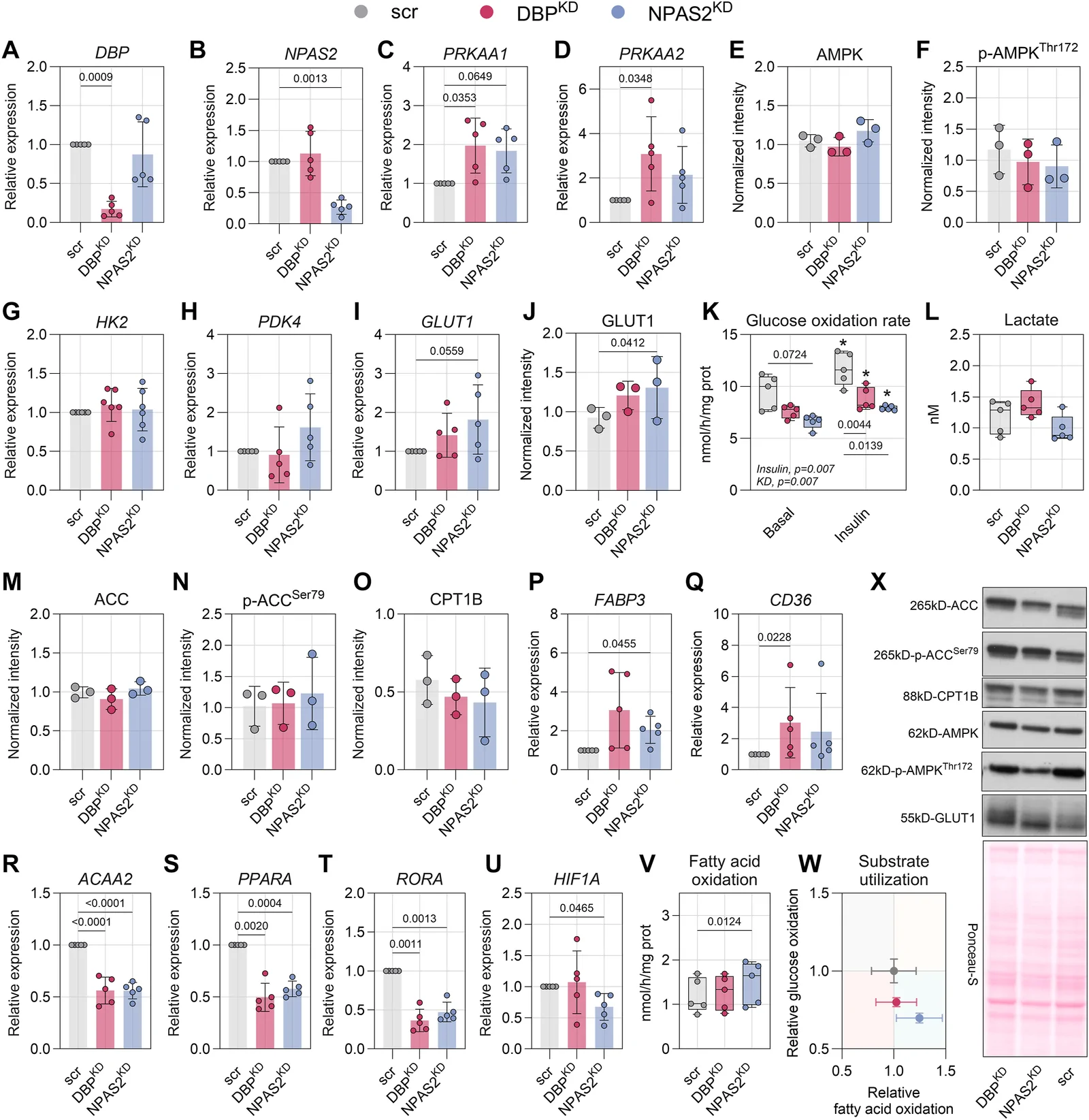
Effects of *DBP* and *NPAS2* silencing on glucose and fatty acid metabolism in human skeletal muscle cells. A-D) Relative gene expression of *DBP* (A), *NPAS2* (B), *PRKAA1* (C), and *PRKAA2* (D). E-F) Protein abundance of AMPK (E), and phospho-AMPK^Thr172^ (F). G-I) Relative gene expression of *HK2* (G), *PDK4* (H), and *SLC2A1* (I). J) Protein abundance of GLUT1. K) Glucose oxidation rates under basal and insulin-stimulated conditions (120 nM). L) Lactate concentration in cell media. M-O) Protein abundance of ACC (M), phospho-ACC^Ser79^ (N) and CPT1B (O). P-U) Relative gene expression of *FABP3* (P), *CD36* (Q), *ACAA2* (R), *PPARA* (S), *RORA* (T), and *HIF1A* (U). V) Palmitate oxidation rate. W) Energy map showing relative glucose and palmitate oxidation across the three conditions. X) Example immunoblots obtained from the same donor across all targets and corresponding Ponceau staining used for intensity normalization. Note that lanes are shown in their original membrane order and therefore do not correspond to the group order used in the graphs. Data from experiments with n > 3 and a single factor were analyzed using one-way repeated-measures ANOVA, followed by Dunnett's test (A-D, G-I, L, P-V). Data from experiments with n = 3 and one factor were analyzed using Friedman's test, followed by Dunn's test (E-F, J, M-O). Data with two factors were analyzed by two-way repeated-measures ANOVA, followed by Tukey's test (K). *P*-values <0.1 are shown on the graphs. ∗, *P* < 0.05 vs corresponding basal condition. Experiments were conducted 48 h post-synchronization.

### 3.4 Effect of gene silencing on mitochondrial function

We next assessed whether the reduction in glucose oxidation reflected impaired mitochondrial function. *NPAS2* silencing increased expression of *PPARGC1A* (Figure 4A), a key regulator of mitochondrial biogenesis. However, this was not accompanied by a corresponding increase in CS abundance (Figure 4B,G). Notably, *NPAS2*-silenced myotubes exhibited reduced abundance of multiple electron transport chain subunits, including complex I (NADH:ubiquinone oxidoreductase subunit A8, NDUFA8), complex III (ubiquinol-cytochrome c reductase core protein 2, UQCRC2), and complex IV (Mitochondrially Encoded Cytochrome C Oxidase II, COXII) components (Figure 4C-E,G), whereas complex V (ATP synthase F1 subunit alpha, ATP5A) remained unchanged (Figure 4F, G). These data suggest that *NPAS2* silencing is associated with selective impairment of respiratory chain composition rather than a global reduction in mitochondrial abundance. To functionally assess these alterations, we performed Seahorse mitochondrial respirometry (Figure 4H). *NPAS2* silencing reduced basal respiration (Figure 4I) and ATP-linked respiration (Figure 4J), consistent with diminished oxidative phosphorylation capacity. In contrast, spare respiratory capacity (Figure 4K), maximal respiration (Figure 4L), and proton leak (Figure 4M) were not significantly altered. Extracellular acidification rate (ECAR) was measured in parallel (Figure 4N). *NPAS2* silencing reduced basal ECAR (Figure 4O) and oligomycin-stimulated ECAR (Figure 4P), whereas ΔECAR (oligomycin-stimulated ECAR – basal ECAR) remained unchanged (Figure 4Q). Because ECAR was obtained during a Mito Stress Test rather than a dedicated Glycolysis Rate Assay, the contribution of glycolytic and respiratory sources to extracellular acidification cannot be determined. Considering lactate production was unchanged (Figure 3L), reduced ECAR may largely reflect reduced respiratory acidification and/or lower overall metabolic activity rather than a clear reduction in glycolytic flux. Combined basal OCR and ECAR measurements are summarized in the energy phenotype map (Figure 4R), illustrating a shift toward a lower basal metabolic flux state without compromising maximal bioenergetic capacity upon *NPAS2* silencing.

**Figure 4:**
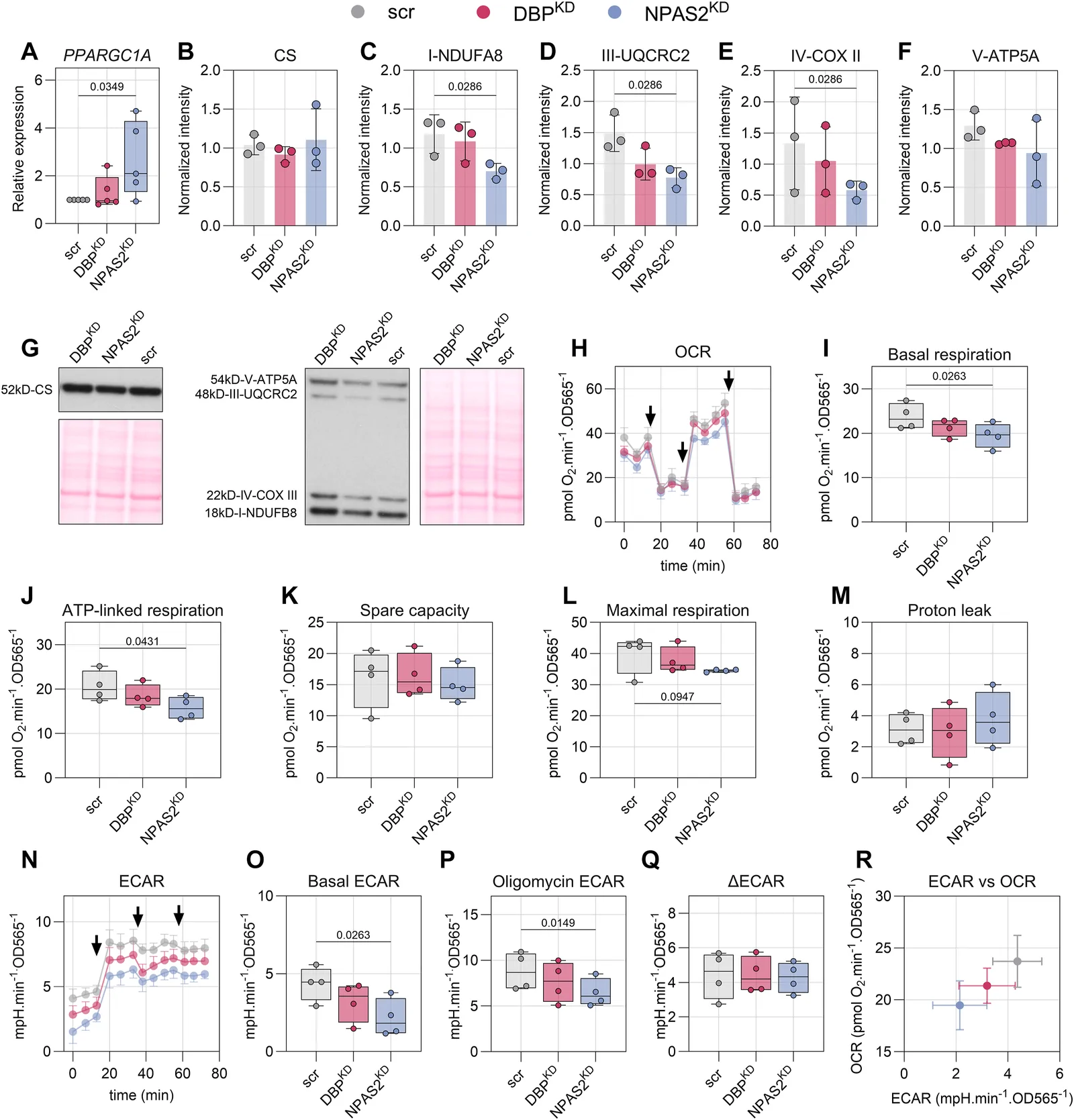
Effects of *DBP* and *NPAS2* silencing on mitochondrial function in human skeletal muscle cells. A) Relative gene expression of *PPARGC1A*. B-F) Protein abundance of CS (B) and mitochondrial electron transport chain subunits I-NDUFA8 (C), III-UQCRC2 (D), IV-COX II (E), and V-ATP5A (F). G) Example immunoblots obtained from the same donor across all targets and corresponding Ponceau staining used for intensity normalization. Note that lanes are shown in their original membrane order and therefore do not correspond to the group order used in the graphs. H) Oxygen consumption rate (OCR) trace measured under basal conditions and following sequential injections of the mitochondrial inhibitors oligomycin, FCCP, and rotenone/antimycin A (arrows), assessed by Seahorse extracellular flux analysis. I-M) Quantification of mitochondrial respiration parameters derived from the OCR trace: basal respiration (I), ATP-linked respiration (J), spare respiratory capacity (K), maximal respiration (L), and proton leak (M). (N) Extracellular acidification rate (ECAR) trace measured under the same conditions of the OCR. O-Q) Quantification of parameters derived from the ECAR trace: basal ECAR (O), oligomycin-stimulated ECAR (P), and ΔECAR (oligomycin-stimulated ECAR – basal ECAR) (Q). R) Energy phenotype map showing OCR versus ECAR, expressed as mean ± SEM. OCR and ECAR values were normalized to protein content. Data from experiments with n > 3 were analyzed using one-way repeated-measures ANOVA, followed by Dunnett's test (A, I-M, O-Q). Data from experiments with n = 3 were analyzed using Friedman's test, followed by Dunn's test (B–F). Experiments were performed 48 h post-synchronization.

## 4 Discussion

AMPK functions as a central cellular energy sensor that coordinates metabolic adaptation to energetic stress and has emerged as an important regulator of circadian timing mechanisms [14,25]. Consistent with our previous observations that pharmacological activation of AMPK modulates clock gene expression in skeletal muscle [16,26], we found that AMPKα silencing alters the expression of core clock genes *CRY1*, *CRY2*, *CLOCK*, and *NPAS2*, as well as the clock-controlled gene *DBP*. Increased *CRY1* and *CRY2* expression is consistent with previous evidence that AMPK regulates CRY stability [15]. Despite these changes in clock gene expression, rhythmicity appeared preserved, suggesting that AMPK influences circadian transcriptional output without disrupting core oscillatory mechanisms. Together, these findings support a role for AMPK in linking energetic status to skeletal muscle circadian gene regulation and identify NPAS2 and DBP as downstream components of AMPK signaling (Figure 5). The reduction in *NPAS2* and *DBP* expression following AMPKα silencing suggests that AMPK-dependent signaling converges on both core and output components of the circadian network. Given that NPAS2 and DBP occupy distinct regulatory levels within this hierarchy, we next asked whether AMPK-regulated clock factors contribute to skeletal muscle metabolic regulation beyond effects on circadian gene expression alone.

**Figure 5:**
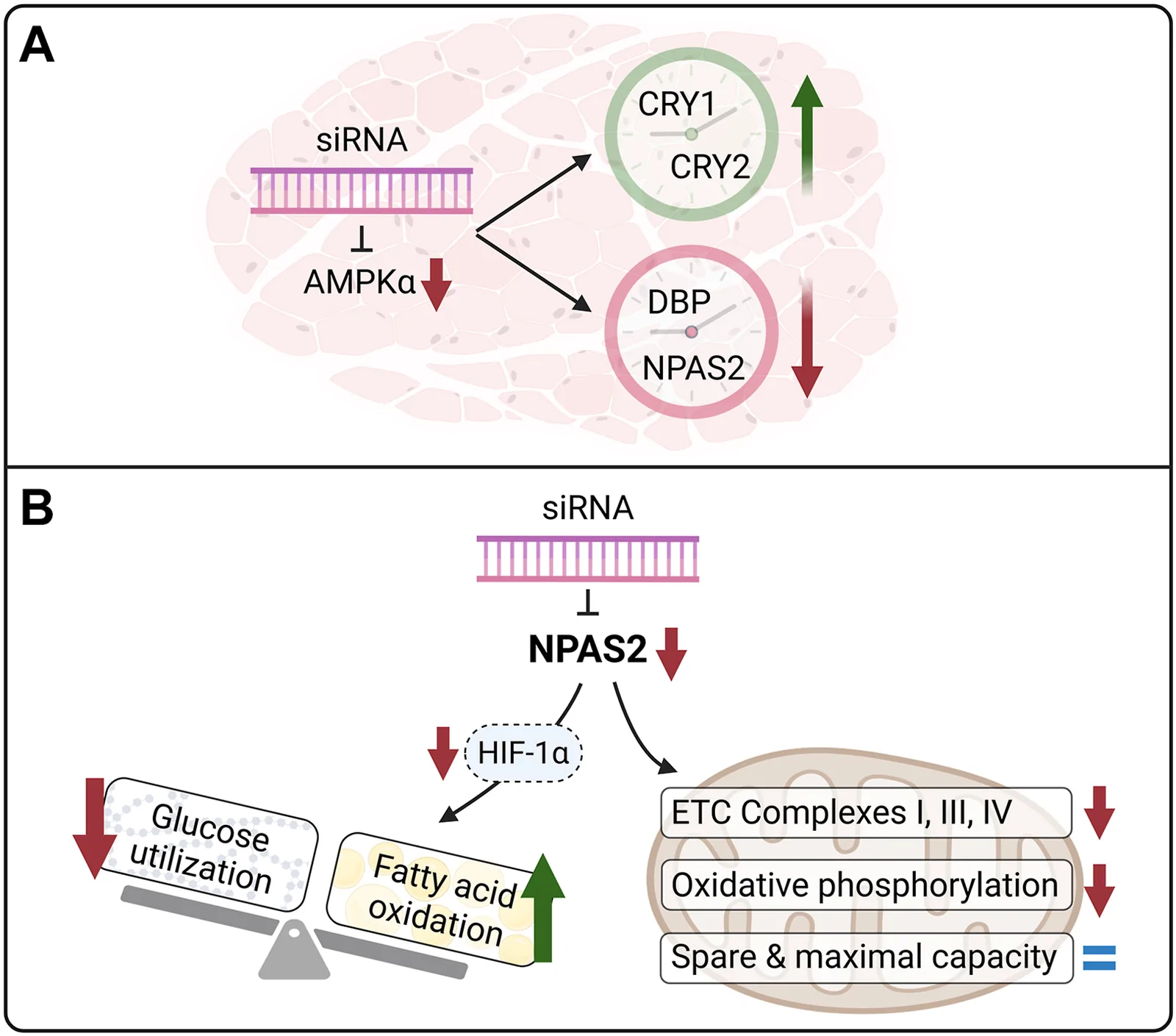
AMPK-dependent regulation of circadian gene expression and metabolic consequences of *NPAS2* silencing in human skeletal muscle cells. A) Silencing of AMPKα in primary human skeletal muscle cells altered expression of circadian genes, increasing expression of the core clock repressors *CRY1* and *CRY2*, while reducing expression of *NPAS2* and the clock-controlled transcription factor *DBP*. B) Proposed model summarizing the metabolic effects of *NPAS2* silencing. Reduction of *NPAS2* expression decreased glucose oxidation while increasing fatty acid utilization. *NPAS2* silencing was additionally associated with reduced abundance of electron transport chain (ETC) complexes I, III, and IV and reduced basal oxidative phosphorylation, whereas spare respiratory capacity and maximal respiratory capacity were preserved. Reduced *HIF1A* expression may represent a link between NPAS2 knockdown and the suppression of glucose metabolism. Collectively, these findings support a role for NPAS2 in coordinating substrate utilization and mitochondrial metabolism in skeletal muscle cells.

NPAS2 is a CLOCK paralog that can compensate for CLOCK deficiency in circadian regulation, whereas combined loss of both CLOCK and NPAS2 disrupts circadian behavior [27]. Although the role of NPAS2 in human skeletal muscle metabolism remains poorly understood, NPAS2 has been reported to promote aerobic glycolysis while suppressing mitochondrial oxidative metabolism in hepatocellular carcinoma cells [24]. Here, *NPAS2* silencing increased *PPARGC1A* expression, suggesting activation of transcriptional programs typically associated with oxidative metabolism and mitochondrial adaptation. However, expression of *RORA*, a known regulator of NPAS2 and lipid metabolism [28], and *ACAA2*, a mitochondrial β-oxidation enzyme, was reduced, indicating broader metabolic transcriptional remodeling rather than a coordinated induction of oxidative pathways. We found that *NPAS2* silencing reduced basal and ATP-linked mitochondrial respiration, accompanied by reduced abundance of multiple electron transport chain subunits and a relative increase in β-oxidation flux. Despite these changes, maximal respiratory capacity was preserved, consistent with preserved mitochondrial oxidative capacity and the presence of excess the electron transport chain capacity [29]. Thus, the increased *PPARGC1A* expression induced by *NPAS2* silencing may represent a compensatory adaptation to the metabolic perturbations associated with NPAS2 deficiency.

The altered substrate utilization and relative increase in fatty acid utilization following *NPAS2* silencing is consistent with the well-established reciprocal relationship between lipid and glucose metabolic pathways, whereby increased fatty acid oxidation is associated with reduced glucose utilization [30]. Consistent with this concept, experimentally induced circadian misalignment in humans promotes fatty acid metabolism gene signatures and reduces skeletal muscle glucose disposal [31], compatible with a potential role for NPAS2 in regulating substrate preference. Alternatively, the reduction in glucose oxidation may reflect the broader suppression of basal oxidative metabolism observed following *NPAS2* silencing, suggesting that impaired mitochondrial utilization of glucose-derived substrates contributes to the phenotype, despite preserved maximal respiratory capacity. In this context, the modest increases in GLUT1 abundance and fatty acid utilization may represent secondary and transient adaptations to reduced oxidative metabolism, rather than primary drivers of the observed metabolic phenotype.

These metabolic alterations were not associated with basal changes in the AMPK-ACC axis, a major regulator of skeletal muscle fatty acid oxidation, nor with altered transcription of the metabolic regulators *HK2* and *PDK4*. Instead, *NPAS2* silencing reduced *HIF1A* expression, suggesting an alternative transcriptional mechanism underlying the observed metabolic phenotype. Disruption of other core clock components, such as BMAL1 and CLOCK, has also been linked to impaired HIF-1α signaling [32,33], supporting the existence of functional interactions between the molecular clock and hypoxia-responsive transcriptional programs. Direct causal testing of this pathway, for example through *HIF1A* knockdown or overexpression, will be necessary to establish this mechanism definitively. HIF-1α is a central transcriptional regulator of glycolytic metabolism that promotes expression of genes involved in glucose uptake and glycolysis while suppressing oxidative metabolism [34]. In skeletal muscle, circadian clock proteins interact with HIF-1α to coordinate glycolytic gene expression and oxygen-dependent metabolism, and disruption of this signaling axis impairs glycolytic metabolic programs [33,35]. Accordingly, reduced *HIF1A* expression may contribute to the diminished glucose utilization observed in *NPAS2*-silenced myotubes.

Silencing of the clock-controlled transcription factor DBP also reduced glucose oxidation. However, *DBP* knockdown did not consistently alter mitochondrial respiration or β-oxidation flux, although directional trends were observed. Together, these findings suggest that the metabolic effects of NPAS2 are not unique among circadian transcriptional regulators expressed in skeletal muscle. Consistent with this interpretation, silencing of *CLOCK* in primary human myotubes reduces glucose uptake, decreases the expression of HIF1-α-target genes, and increases the expression of genes involved in lipid handling and fatty acid metabolism [32]. These findings support a broader role for the skeletal muscle clock in regulating substrate utilization and metabolic homeostasis.

Collectively, NPAS2 appears to contribute to the coordination of substrate utilization and mitochondrial metabolism in skeletal muscle. *NPAS2* silencing was associated with preserved maximal respiratory capacity, reduced basal oxidative metabolism, increased β-oxidation flux, and altered respiratory chain composition. These findings suggest a role for NPAS2 in integrating the core clock machinery with skeletal muscle metabolic regulation. Although NPAS2 and DBP are circadian regulators, we did not assess clock gene rhythmicity following silencing of these genes. Therefore, we cannot determine whether the observed metabolic phenotypes arise from altered circadian function.

In conclusion, these findings support the concept that molecular clock components are active regulators of skeletal muscle metabolic phenotype rather than passive timekeepers. Given the well-established association between circadian disruption and metabolic disease [36,37], a deeper understanding of the molecular pathways linking peripheral clocks to substrate utilization and mitochondrial function may reveal new opportunities to improve metabolic health. Further studies are warranted to determine whether targeting components of the AMPK–circadian clock axis can mitigate metabolic dysfunction associated with circadian disruption.

### 4.1 Limitations

A limitation of this study is the use of cultured primary human myotubes, which do not fully recapitulate in vivo endocrine, neural, and behavioral regulation of metabolism. Moreover, only male donor-derived myotubes were studied. Given established sex differences in skeletal muscle metabolism, potential sex-specific effects on circadian regulation and metabolic responses could not be assessed. In addition, metabolic flexibility was inferred from basal measurements rather than directly assessed using dynamic substrate-switching experiments. Additionally, the molecular mechanisms linking AMPK, NPAS2, HIF-1α, and glycolytic metabolism were beyond the scope of the present work but warrant further investigation. Finally, although *DBP* silencing produced effects on glucose oxidation, the limited sample size may have reduced sensitivity to detect additional metabolic changes.

## CRediT authorship contribution statement

**David Rizo-Roca:** Conceptualization, Formal analysis, Visualization, Writing – review & editing. **Logan A. Pendergrast:** Conceptualization, Investigation, Methodology, Formal analysis, Writing – original draft, Writing – review & editing, Visualization. **Prasad Kamble:** Conceptualization, Investigation, Methodology, Writing – review & editing. **Dimitrius Santiago P.S.F. Guimarães:** Investigation, Writing – review & editing, Validation, Methodology. **Lucile Dollet:** Conceptualization, Writing – review & editing, Supervision. **Marie Björnholm:** Writing – review & editing, Supervision, Project administration. **Juleen R. Zierath:** Writing – review & editing, Writing – original draft, Supervision, Resources, Project administration, Funding acquisition, Conceptualization.

## Ethics declaration

Written informed consent to take part in the study and to publish the article has been obtained from all participants or their legal representatives. The privacy rights of participants have been observed.

This study included organ or tissue donors. This study includes human biological material and consent was obtained by donors, or their next of kin or legal representatives, for use in this study and for publication of the article. The samples used in this research were not sourced from executed prisoners or prisoners of conscience.

This study was performed in compliance with relevant laws, regulatory frameworks and guidelines where the research took place. This study was approved by the Regional Ethical Review Board in Stockholm. (Approval No. (2012-1955-31-1 and 2012-1047-31-2))

## Funding

This study was supported by the 10.13039/501100009708Novo Nordisk Foundation (NNF14OC0011493), Swedish Diabetes Foundation (DIA2023-824), 10.13039/501100001648EFSD/10.13039/501100004191Novo Nordisk A/S Programme in Diabetes Research in Europe 2021 and 10.13039/501100001648EFSD/Lilly European Diabetes Research Programme 2023, the 10.13039/501100004359Swedish Research Council (2015-00165), the 10.13039/501100000781European Research Council (ERC-2023-AdG 101142093) and a Wallenberg Scholar grant from the 10.13039/501100004063Knut and Alice Wallenberg Foundation (2023.0312). D. R–R holds an Excellence Emerging Investigator Grant from 10.13039/501100004191Novo Nordisk Foundation (NNF25OC0101637). L.A.P. was supported by KID funding from 10.13039/501100004047Karolinska Institutet (2019). D.S.P.S.F.G was supported by the 10.13039/501100000780European Commission 10.13039/501100007601Horizon 2020 research and innovation programme under the Marie Skłodowska-Curie (101,146,767). The 10.13039/501100011747Novo Nordisk Foundation Center for Basic Metabolic Research (CBMR) is an independent research center at the 10.13039/501100006442Faculty of Health and Medical Sciences, University of Copenhagen, Denmark, partially funded by an unrestricted donation from the 10.13039/501100009708Novo Nordisk Foundation (NNF18CC0034900, NNF23SA0084103).

## Declaration of competing interest

The authors declare that they have no known competing financial interests or personal relationships that could have appeared to influence the work reported in this paper.

## Data Availability

Data will be made available on request.
