# Supplementary material for: The circadian clock component NPAS2 is modulated by AMPK and regulates substrate utilization and mitochondrial function in human skeletal muscle cells
Source: Mol Metab. 2026 Sep 19;113:102446. doi: 10.1016/j.molmet.2026.102446 (PMC13653946; doi:10.1016/j.molmet.2026.102446)
Supplement: Supplementary file 1 [file mmc1.docx]

**Figure S1.** a) Expression of the myogenic markers desmin (*DES*), myosin heavy chain 2 (*MYH2*), myogenin (*MYOG*), myogenic factor 5 (*MYF5*), myoblast determination protein 1 (MYOD1), and paired box protein Pax-7 (*PAX7*), assessed by qPCR in myotubes 12 days after induction of differentiation. The obtained C_T_ values are consistent with a differentiated myotube phenotype.
